# SmartEye and *Polhemus* data for *vestibulo–ocular reflex* and *optokinetic reflex* model

**DOI:** 10.1016/j.dib.2018.03.097

**Published:** 2018-03-27

**Authors:** Anh Son Le, Hirofumi Aoki

**Affiliations:** a Human Factors and Aging Laboratory, Institutes of Innovation for Future Society, Nagoya University, Nagoya, Japan; b Department of Power Engineering, Faculty of Engineering, Vietnam National University of Agriculture, Hanoi, Vietnam

**Keywords:** Driver distraction, Vestibulo–ocular reflex, Optokinetic reflex

## Abstract

In this data article, this dataset included raw data of head and eye movement that collected by Polhemus (Polhemus Inc) and SmartEye (Smart Eye AB) equipment. Subjects who have driver license participated in this experiment. The experiment was conducted with a driving simulator that was controlled by CarSim (Mechanical simulation Co., Anna Arbor, MI) with the vehicle motion. This data set not only contained the eye and head movement but also had eye gaze, pupil diameter, saccades, and so on. It can be used for the parameter identification of the vestibulor-ocular reflex (VOR) model, simulation eye movement, as well as running other analysis related to eye movement.

**Specifications Table**

**Table:** 

Subject area	Psychology, transportation
More specific subject area	Driver distraction evaluation
Type of data	Table, log file, CSV file, video file.
How data was acquired	Real-time head and eye movement recording
Data format	the log file, CSV file
Experimental factors	Eye simulation based on the head measurement
Experimental features	A participant was drove following the design course thrice: drove without visual stimulus, drove with visual stimulus, and drove with visual stimulus and mental workload (detail in experimental setup part).
Data source location	Institute of Innovation for Future Society, Nagoya University, Furo-cho, Chikusa-ku, Nagoya, 464–8601, Japan
*Data accessibility*	Data available within this article

**Value of the data**

• Parameter identification for VOR model.
• Parameter identification for optokinetic (OKR) model.
• Data provides the possibility for analyzing the effect of visual information on eye movement.
• Data also provides the information of eye movement while driving with the mental workload.

## 1 Data

+ Smart Eye data: data of eye tracking used Smart Eye equipment [1] (log file).

The Smart Eye data was collected with a 120 Hz sampling rate that included head tracking, eye position, eye gaze, pupil diameter, saccades, fixations, and many more.

+ Polhemus data: Head movement measurement.

The Polhemus equipment was recorded head movement data with the sampling rate (60 Hz). This data contained head position and velocity that can use for inputting of VOR model or OKR model.

## 2 Experimental design, materials and methods

**Fig. 1:**
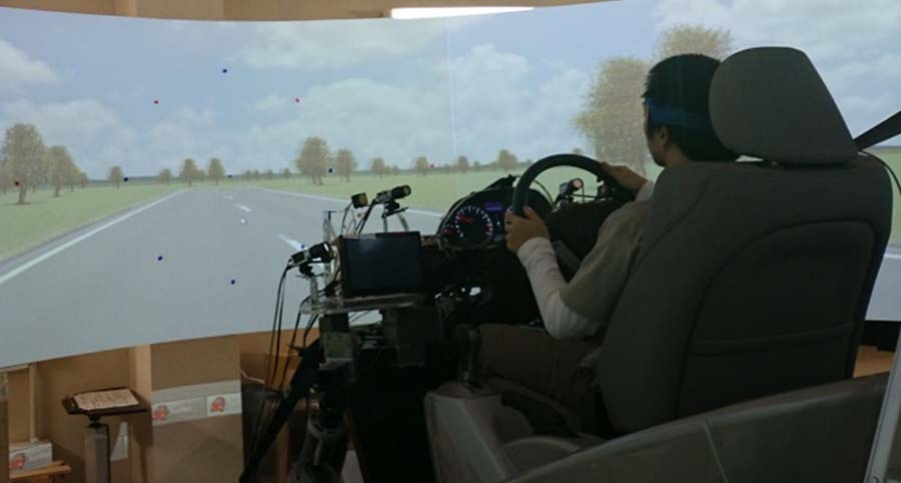
Overview of the experimental setup.

In the experiment, each subject was asked to drive around a simulated course while seated in a driving simulator with six degrees of freedom. The simulator was controlled by CarSim, which can simulate the dynamic behavior of a vehicle (Fig. 1). In these experiments, the seat was moved with a fixed frequency in the vertical and horizontal plane by using MATLAB Simulink (MathWorks, Natick, MA) to control CarSim.

A subject who held drivers’ licenses participated in the experiment. Each participant followed the course three times: without Visual Stimulus, with Visual Stimulus, and with Visual Stimulus and Mental Workload.

• Visual stimulus (VS): Simulated trees were positioned alongside the test track in the driving simulator to help induce large quantities of optical flow.
• Driving without VS: The subject was asked to drive around a designed course without any simulated objects alongside the road.
• Driving with VS: The subject was asked to drive around the same course with simulated trees along the road.
• Driving with VS and the n-back task: The subject was asked to drive around the same course with simulated trees alongside the road while performing a one-back task within two seconds by pressing appropriate buttons on the steering wheel.

This data can be used to simulate eye movement based on head movement such as [2], [3], Obinata group [4], [5], [6], [7], [8], [9], Anh Son et al. [10], [11], [12], [13], [14], [15], and so on. In addition, this data can use to see the effect of a visual stimulus or mental workload on driver performance as well as eye movement.
