# Supplementary material for: SmartEye and Polhemus data for vestibulo–ocular reflex and optokinetic reflex model
Source: Data Brief. 2018 Mar 27;18:882–5. doi: 10.1016/j.dib.2018.03.097 (PMC5996827; doi:10.1016/j.dib.2018.03.097)
Supplement: Supplementary file 1 — Supplementary material [file mmc1.pdf]

Conflicts of Interest Statement

Manuscript title: SmartEye and Polhemus data for vestibulo–ocular reflex and optokinetic reflex model

- ☑ All authors have participated in (a) conception and design, or analysis and interpretation of the data; (b) drafting the article or revising it critically for important intellectual content; and (c) approval of the final version.
- ☑ The Article I have submitted to the journal for review is original, has been written by the stated authors and has not been published elsewhere.
- ☑ The Images that I have submitted to the journal for review are original, was taken by the stated authors, and has not been published elsewhere.
- ☑ This manuscript has not been submitted to, nor is under review at, another journal or other publishing venue.
- ☑ The authors have no affiliation with any organization with a direct or indirect financial interest in the subject matter discussed in the manuscript
- ☑ The below authors have affiliations with organizations with direct or indirect financial interest in the subject matter discussed in the manuscript:

This statement is signed by all the authors to indicate agreement that the above information is true and correct.

Author's name	Author's signature	Date
LE ANH SON	[signature]	19/3/2018
HIROFUMI AOKI	[signature]	19/3/2018
